# Interactions among salt marsh plants vary geographically but not latitudinally along the California coast

**DOI:** 10.1002/ece3.3191

**Published:** 2017-07-14

**Authors:** Akana E. Noto, Jonathan B. Shurin

**Affiliations:** ^1^ Section of Ecology, Behavior and Evolution University of California, San Diego La Jolla CA USA

**Keywords:** biogeography, community ecology, competition, facilitation, stress‐gradient hypothesis

## Abstract

The strength of species interactions often varies geographically and locally with environmental conditions. Competitive interactions are predicted to be stronger in benign environments while facilitation is expected to be stronger in harsh ones. We tested these ideas with an aboveground neighbor removal experiment at six salt marshes along the California coast. We determined the effect of removals of either the dominant species, *Salicornia pacifica*, or the subordinate species on plant cover, aboveground biomass and community composition, as well as soil salinity and moisture. We found that *S. pacifica* consistently competed with the subordinate species and that the strength of competition varied among sites. In contrast with other studies showing that dominant species facilitate subordinates by moderating physical stress, here the subordinate species facilitated *S. pacifica* shortly after removal treatments were imposed, but the effect disappeared over time. Contrary to expectations based on patterns observed in east coast salt marshes, we did not see patterns in species interactions in relation to latitude, climate, or soil edaphic characteristics. Our results suggest that variation in interactions among salt marsh plants may be influenced by local‐scale site differences such as nutrients more than broad latitudinal gradients.

## 1 INTRODUCTION

Species interactions often vary geographically. For instance, stronger consumption of invertebrates and plants has been shown at low latitudes in marine and salt marsh communities, respectively (Freestone, Osman, Ruiz, & Torchin, 2011; Kimbro et al., 2014; Pennings et al., 2009). These trends are attributed in part to increased productivity and diversity at low latitudes and subsequently stronger interactions among species (MacArthur 1972; Schall & Pianka, 1978; Stachowicz & Hay, 2000; Hillebrand, 2004; Pennings & Silliman, 2005; Pennings et al., 2009). However, species interactions may also be shaped by local‐scale environmental variation that does not follow clear latitudinal patterns (Feller et al., 2013; Pennings, Selig, Houser, & Bertness, 2003). Geographic patterns in species interactions and the mechanisms driving them are informative for understanding the functioning of ecological systems and predicting how communities will change with the environment.

The stress‐gradient hypothesis (SGH) is one of the dominant paradigms for understanding how interactions among plants change across physical and biological stress gradients. The SGH posits that facilitative interactions dominate in harsh environments where neighboring plants moderate stresses such as desiccation or heat, whereas competitive interactions are more prevalent in benign environments (Bertness & Callaway, 1994). For instance, in salt marshes, plants can facilitate others by creating cool, moist, low‐salinity microclimates (Callaway, 1994). Consistent with this hypothesis, studies across ecosystems have shown that competition is stronger where temperatures are warm and precipitation is abundant (Callaway et al., 2002; Cavieres & Badano, 2009; Tewksbury & Lloyd, 2001). Similar trends occur across soil nutrient, salinity, and inundation (anoxia) gradients, indicating that the SGH applies to abiotic factors other than temperature and precipitation (Bertness & Ewanchuk, 2002; Bertness & Hacker, 1994; Espeland & Rice, 2007).

Facilitation is often a result of dominant foundation species that affect the community by shaping the local environment (Bertness & Callaway, 1994; Bruno & Bertness, 2001; Ellison et al., 2005; Stachowicz, 2001). These species create refuges from predation or environmental stress by mechanisms such as providing structure or altering microclimates (Altieri, Silliman, & Bertness, 2007; Bruno & Bertness, 2001; Callaway, 1994; Ellison et al., 2005). Yet in some cases, subordinate species facilitate the dominant species. For example, in a desert community a dominant annual grass was only able to recover from a disturbance in the presence of the subordinate species (Boeken & Shachak, 2006; Grime, 1998). Frequently, each species exerts both a facilitative and competitive effect on the other, and which effect dominates depends on plant traits and the environment (He, Bertness, & Altieri, 2013; Morzaria‐Luna & Zedler, 2014). Thus, the strength of interactions between dominant and subordinate species may vary in space depending on the environment and the mechanisms by which each shapes the environment.

Geographic shifts in species interactions with the environment may depend on the spatial scale of comparison and what is considered stressful for a particular species (He & Bertness, 2014). In alpine plants, facilitation increased with environmental stress over short distances, but decreased again as environmental stress intensified when the gradient was extended (Cavieres & Badano, 2009). Salt marsh plants in southern New England, where soil salinity is high, facilitate each other more than in low‐salinity northern New England, as predicted by the SGH (Bertness & Ewanchuk, 2002). Yet interactions are equally competitive in salt marshes in New England and the south Atlantic US coast despite greater salinity in the south (Pennings et al., 2003). The SGH may show scale dependence as local adaptation and species turnover among distant sites may produce communities that are adapted to more stressful conditions and are not stressed by them (He & Bertness, 2014; He et al., 2013; Pennings et al., 2003). As a result, tests of the SGH over a broad geographic and environmental gradient containing the same species are particularly informative as they show how species interactions change over large areas without species turnover.

Variation in interactions among plants has seldom been tested in west coast marshes across a large geographic scale. Along an estuarine gradient in Oregon, interactions among plants were more competitive at lower salinities as expected by the SGH, although unlike in east coast marshes, facilitative effects of neighbors were rarely observed (Keammerer & Hacker, 2013). This test was only within one site, and few studies have examined the variation in interaction strength among multiple sites along a gradient rather than at two ends of it. In addition, stress in intertidal habitats on the west coast may not show the same clear latitudinal patterns as on the east coast due to the importance of microclimates and variation in timing of high temperatures and tidal exposure (Helmuth et al., 2002, 2006). A larger, more finely resolved gradient may allow us to better understand how the environment affects species interactions when climatic patterns are not clearly latitudinal.

We conducted a neighbor removal experiment in six sites spanning 8° of latitude on the California coast to determine how interactions among salt marsh plant species vary geographically. An improved understanding of the drivers of species interactions across space will allow us to better predict how species interactions will be affected by climate change. This can inform conservation efforts which is particularly important in salt marshes as they are already highly threatened (Gedan, Silliman, & Bertness, 2009; UNEP 2006). Temperature and precipitation both vary latitudinally, so we hypothesized that interaction strength and abiotic variables such as soil salinity would also vary among sites. Reduced salinity is typically associated with greater competition among sites on the east coast (Bertness & Ewanchuk, 2002) and within sites on the west coast (Pennings & Callaway, 1992). Thus, we hypothesized that interactions would be more competitive in the north where low temperatures and abundant precipitation are likely to lead to low salinity. Facilitation of subordinate species by the dominant may be expected in the south where high temperatures and low precipitation lead to high salinity.

## 2 MATERIALS AND METHODS

### 2.1 Study system

Plots were selected in the mid‐marsh in six California salt marshes spanning approximately 1100 km and 8° latitude (Fig. S1). Sites from south to north were in the Tijuana Estuary (TJ), Kendall‐Frost Mission Bay Marsh Reserve (KF), Carpinteria Salt Marsh Reserve (CAR), Elkhorn Slough (ELK), Tomales Bay (TOM), and Humboldt Bay (HUM; Fig. S1). Sites span a fourfold gradient in precipitation and a 6°C difference in mean temperature (Fig. S1; Arguez et al. 2012). However, there was little species turnover among sites as an average of 84% of vegetation cover was composed of five species that were present across the entire range (*Salicornia pacifica*,* Jaumea carnosa*,* Distichlis spicata*,* Limonium californicum*,* Triglochin concinna*) out of 13 recorded species (Table S1). As northern sites tended to face directly onto bays (HUM, TOM, ELK) while southern sites were generally estuarine (CAR, TJ), we included two sites in San Diego, one on a bay (KF) and the other on an estuary (TJ), to ensure that marshes on bays were included among both northern and southern sites. This was necessary as differences in exposure between bays and estuaries may contribute to differences in environmental factors such as wave energy and inundation which could affect soil salinity or moisture via differing soil temperature and evaporation patterns (Helmuth et al., 2006). Finally, tidal range varied among sites with larger tidal ranges generally in the north.

This experiment focused on the effect of a common salt marsh species, *S. pacifica* (formerly *Salicornia virginica*), on the surrounding plant community. *S. pacifica* is a dominant species in marshes on the eastern coast of the Pacific from Baja California to Canada as it occurs in great abundance and is competitively superior to other species (Macdonald & Barbour, 1974). It is a perennial succulent forb that grows upright, reproduces both vegetatively and from seed, and occurs across much of the intertidal zone (Sullivan and Noe 2001). Subordinate species in these marshes are mainly perennial forbs and grasses and include *J. carnosa*,* D. spicata*, and *Frankenia salina* (Table S1). These species occur in most marshes but occur in a narrower range of conditions and can be slower growing and weaker competitors than *S. pacifica* (Armitage, Boyer, Vance, & Ambrose, 2006; Bonin & Zedler, 2008; Parker et al., 2011; Zedler, 1977). Plots were selected to contain *S. pacifica* and other species, including at least *J. carnosa*. The conditions under which *S. pacifica* co‐occurs with other species vary across sites, so plots were not at identical elevations but were similar distances from channels, except at Elkhorn Slough where biological conditions required plots to be closer to channels.

### 2.2 Experimental design

In fall 2013, we established an aboveground neighbor removal experiment to determine how aboveground species interactions differ across this gradient. At each site, we established three treatments in 1 m × 1 m plots replicated five times: *S. pacifica* removal, subordinate plant removal, and no‐removal control plots. *S. pacifica* removals determined the effect of *S. pacifica* on the surrounding plant community, while subordinate species removal measured the effect of associated species on *S. pacifica*. Initial percent cover of each species was visually estimated in each plot as the percentage of the plot covered by that species. Percent cover estimates accounted for layering such that total cover could exceed 100%. Blocks of three plots with similar starting compositions were established, and 100% of *S. pacifica* cover was removed from *S. pacifica* removal plots. An equivalent amount of cover was removed from the blocked subordinate species removal plot; for example, if *S. pacifica* cover was 50%, 50% cover of subordinate species was removed from subordinate removal plots. On average, 54% cover was removed from removal plots. No plant cover was removed from the control plot in each block. Plants were removed by clipping aboveground biomass at the soil surface, and plots were checked every 3 months and any vegetation regrowth was removed. Typically, no more than 1%–2% cover of *S. pacifica* grew back.

Percent cover in plots was sampled for 2 years in late March, when annual plants start to grow, and late September, when biomass peaks. There was only one annual plant species, *Salicornia bigelovii*, so it was unlikely to have a very large impact. At the end of the experiment in September 2015, we collected aboveground biomass in a 0.1 m × 1 m strip of the plot 0.1 m from the edge of the plot. Biomass was brought back to the laboratory, sorted to species, and dried at 40°C to constant weight.

We made several measurements to characterize environmental differences among plots and sites. We collected 2‐cm‐diameter soil cores from the soil surface in the center of each plot which we used to determine soil moisture and soil salinity. Soil cores were collected in spring and fall at low tide. Soil moisture was measured as percent weight loss when samples were dried at 100°C for 24 hr. To measure salinity, dried soil samples were homogenized, deionized water was added until soils were saturated, and porewater was squeezed through a Whatman number 3 qualitative grade filter onto a refractometer (Callaway 2012). Finally, we used magnesium calcite chalk blocks (dental chalk) to compare wave energy among plots and sites (Bertness, Gaines, Bermudez, & Sanford, 1991; Bertness et al., 2014). Blocks were weighed, attached to wire mesh, and deployed in the field in fall 2014. After 14 weeks, chalk blocks were brought back to the laboratory, gently rinsed, and dried at 40°C to constant weight. Chalk loss per day measures erosion and was used as an indication of wave energy.

### 2.3 Statistical analyses

Differences among sites and removal treatments in plant cover, aboveground biomass, and species richness were determined using linear mixed‐effects models. Block was included as a random factor in these models, and time was included as a fixed factor to account for having measured plant cover four times after the experiment was established. Time was not included in biomass models as biomass was only measured at the end of the experiment. The effects of site and removal treatment on plant community composition were determined using distance‐based redundancy analyses (dbRDA) in the vegan package in R (Oksanen et al., 2015). We used Bray–Curtis dissimilarities as they do not include shared zeroes.

Interaction strengths were assessed using the log response ratio, a comparison of paired removal and control plots (Hedges, Gurevitch, & Curtis, 1999; Pugnaire & Luque, 2001). The effect of subordinate species on *S. pacifica* was calculated within a block as ln(*S. pacifica* in control/*S. pacifica* without subordinate species). The effect of *S. pacifica* on subordinate species was calculated as ln(subordinate species in control/subordinate species without *S. pacifica*). Positive interaction strengths indicate facilitation while a negative interaction strength indicates competition. Interaction strengths were calculated for both percent cover and biomass. Differences among sites were evaluated using fixed‐effect models (including time in the case of cover). Block was not included as a factor in this case as calculations of interaction strength already group blocked plots together. The subordinate species effect on *S. pacifica* did not differ by site, so we aggregated data from all sites and conducted one‐sample *t*‐tests to determine whether interaction strengths at each sampling date differed significantly from zero. We applied a Bonferroni correction to account for repeated *t*‐tests at several time points. Finally, we conducted a power analysis to ensure that our sample size was sufficiently large to detect latitudinal or climate‐related variation in interaction strength (power = 0.8, alpha = 0.05).

Variation in environmental variables was assessed using fixed‐effect models with site and removal treatment as fixed factors. We used linear regressions to test for relationships between interaction strength and latitude, site‐level mean precipitation, and mean temperature for which we obtained data from NOAA. We also used linear regression to test for relationships between interaction strengths and all measured local environmental variables (salinity, soil moisture, and wave energy). Because each interaction strength was calculated based on paired plots (control and removal), local environmental analyses compared interaction strength to the average environmental measure in those same plots. All analyses were performed in R v. 3.2.0 (R Development Core Team 2015).

## 3 RESULTS

**Figure 1:**
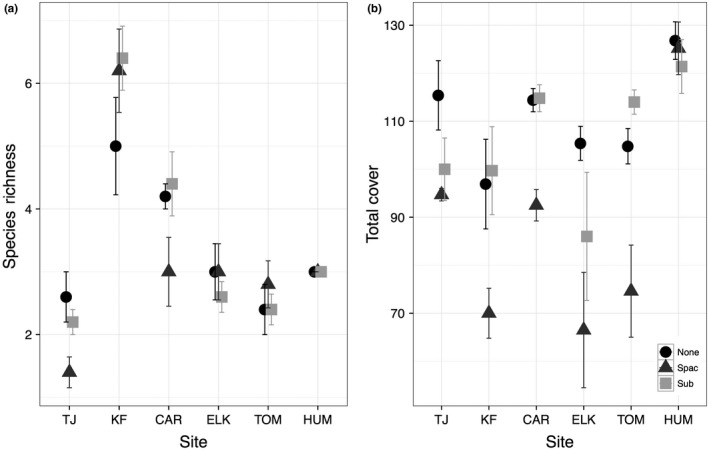
(a) Species richness and (b) total cover averaged across all time points in control (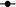), *Salicornia pacifica* removal (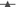), and subordinate species removal (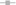) plots. Sites are listed in order of latitude, south to north. Values are means ± *SE*

Plant communities differed substantially among sites. Total cover and biomass, subordinate species cover, and species richness varied significantly by site but did not follow latitudinal patterns (Figure 1; Table 1; Table S2). Rather, sites that were closest together often varied considerably (Figure 1). Total cover was significantly reduced by both *S. pacifica* and subordinate species removals (Tukey: *p* < .001 comparing either treatment to control; Table S2), although the effect of subordinate species removal varied among sites and was more apparent at some sites than others (Figure 1). The effect of subordinate species removal also weakened over time; while those plots initially differed in cover from control plots (Tukey: *p* < .001), cover later became comparable to control plots at several sites (Figure 1). Subordinate species cover was also reduced by its removal (Tukey: *p* < .001 compared to control; Table S2), indicating that removal treatments were successful. However, total and subordinate species cover and richness increased over time after initial removals (Table 1).

**Table 1:** *F*‐values from mixed‐effects models of plant community metrics. Analyses were performed on cover and biomass of all species as well as only subordinate species. Biomass was sampled only once, so time was not included as a factor in that analysis

	Site (*S*)	Removal (*R*)	Time (*T*)	*R *× *S*	*R *× *T*	*S *× *T*	*R *× *S *× *T*
*df*	5	2	3	10	6	15	30
Total cover	16.17***	179.16***	38.82***	3.54**	10.77***	3.57***	1.73**
Subspecies cover	16.75***	96.32***	19.72***	5.91***	1.02	6.83***	1.65*
Richness	18.00***	1.79	5.42**	6.98***	0.15	0.88	0.38
Subspecies richness	17.84***	0.39	3.23*	5.87**	0.93	0.83	0.24
Total biomass	5.10**	29.90***	–	1.43	–	–	–

*p < .05, **p < .01, ***p < .001.

**Figure 2:**
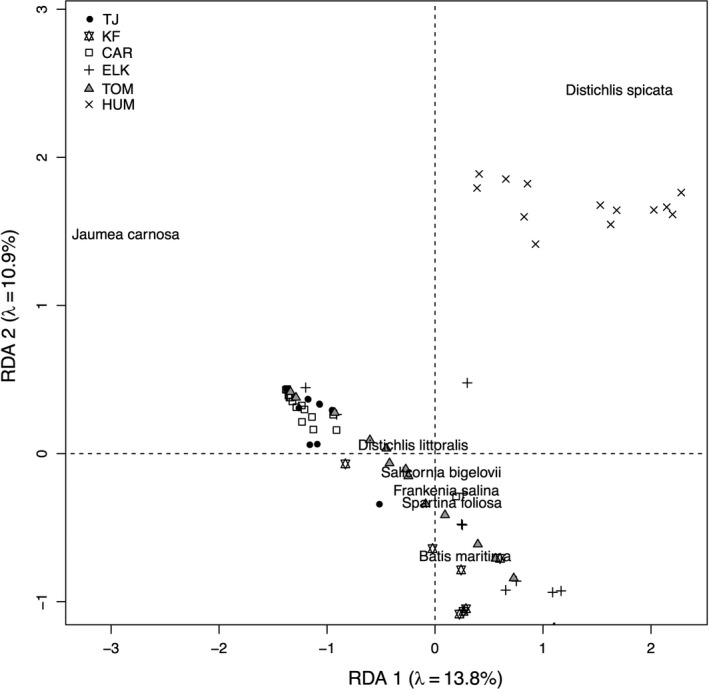
Distance‐based redundancy analysis excluding *S. pacifica* from analysis. Only species with scores >0.1 were included in the figure

Sites also differed in their community composition, but unlike other community‐level measures, community composition showed a significant latitudinal trend (dbRDA latitude effect: *F*_1,84_ = 18.79, *p* < .001). Humboldt Bay is distinct in composition from the other sites as it is heavily dominated by *D. spicata*, while other sites show substantial overlap in composition with two northern sites, Tomales Bay and Elkhorn Slough, and two southern sites, Tijuana Estuary and Carpinteria, clustering together (Figure 2).

**Figure 3:**
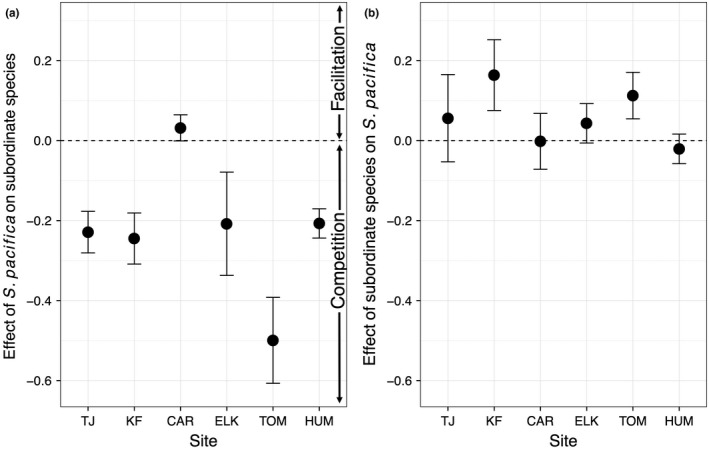
Interaction strengths based on cover depicting the effect of (a) *S. pacifica* on subordinate species and (b) subordinate species on *S. pacifica*. Sites are listed in order of latitude, south to north. Values are means ± *SE*

We also examined geographic differences in interaction strengths. The effect of subordinate species on *S. pacifica* was consistent among sites (F_5,96_ = 0.95, *p* = .45). On average, subordinate species significantly facilitated *S. pacifica* (*t*_119_ = 1.98, *p* = .05), although this was due to facilitation at the initial sampling date with no significant effect at later sampling dates (Figure 3, Fig. S2; Spring 2014: *t*_29_ = 3.75, *p* < .001, α_corrected_ = 0.0125). This suggests that the sampling time frame was sufficient to observe effects of the removal treatment, although the facilitative effect of subordinate species weakened over time.

*S. pacifica* removal treatments revealed that the dominant species generally had a competitive effect on the subordinate species (Figure 3, Fig. S2). The effect of *S. pacifica* removal on subordinate species cover varied by site (Table 1; *F*_5,96_ = 4.24, *p* = .0016). For instance, Carpinteria, a central site, consistently showed slight facilitation (i.e., the weakest competition) and Tomales Bay, a northern site, always showed the strongest competition (Figure 3, Fig. S2). When we calculated interaction strengths based on biomass measured at the end of the experiment rather than cover, however, neither group had a significant effect on the other (*S. pacifica* on sub: *t*_29_ = 1.35, *p* = .19; sub on *S. pacifica*:* t*_29_ = 0.21, *p* = .84). Despite geographic differences in interactions strengths based on cover, the effect of *S. pacifica* on subordinate species was not correlated with latitude (*r* ^2^ = .015, *p* = .19), mean temperature (*r* ^2^ = .16, *p* = .24), or mean precipitation (*r* ^2^ = .093, *p* = .29). A power analysis indicated that 4 × 10^8^ samples would be required to produce a significant relationship between interaction strength and latitude based on the slope and standard deviations that we obtained in our regression, and temperature and precipitation would require similarly large sample sizes to produce significant relationships.

**Figure 4:**
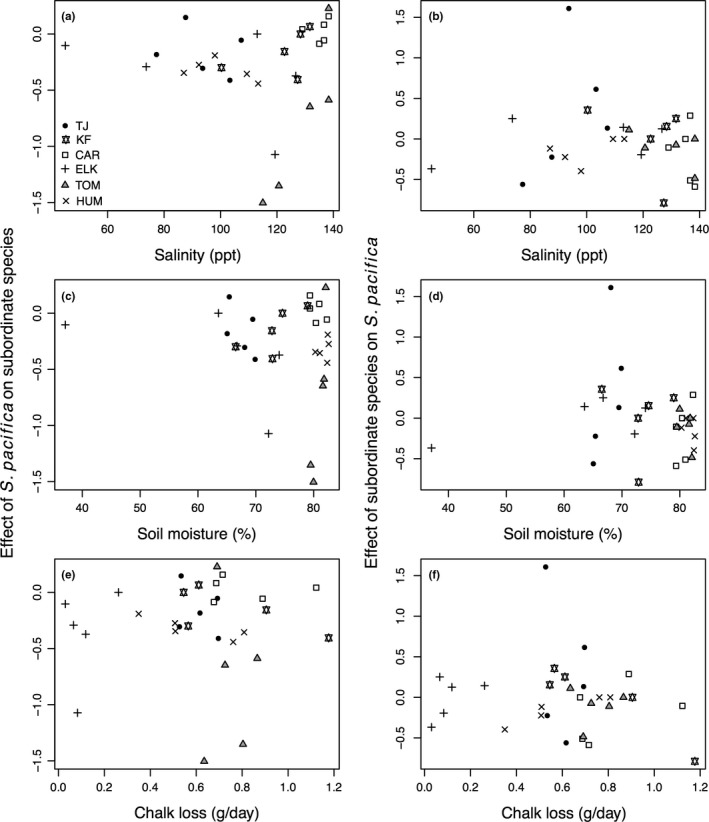
Interaction strengths based on cover at each site in relation to salinity (parts per thousand; a,b), soil moisture (percentage; c,d), and wave energy as measured by mass of chalk lost per day (g/day; e,f). All environmental values are averages of values measured in the plots used to calculate interaction strength. No relationships were significant

We also considered local environmental variables and found significant differences among sites in soil salinity, soil moisture, and wave energy (Table 2; Figs. S1, S3). Removal treatments did not significantly affect these measures, suggesting that neither *S. pacifica* nor subordinate species have substantial effects on these aspects of the environment. Measured environmental conditions also did not vary latitudinally with, for instance, the weakest wave energy at centrally located Elkhorn Slough (Figs. S1, S3). Finally, we investigated the relationship between local environmental variables and interaction strengths and found no significant relationships (Figure 4).

**Table 2:** *F*‐values from ANOVA results for local‐scale environmental variables

	Site	Removal	Site* *× removal
*df*	5	2	10
Wave energy	17.85***	2.16	0.694
Salinity	9.03***	0.169	0.448
Soil moisture	18.38***	0.552	0.264

***p < .001.

## 4 DISCUSSION

We found differences in plant communities and interactions among sites, but these largely did not follow latitudinal trends. Geographic differences in cover, richness, and interaction strength were unrelated to latitude or large‐scale climatic variables. The competitive effect of the dominant species, *S. pacifica*, on subordinate species varied among sites, but was unrelated to latitude, climate, or any of several measured local environmental conditions (Figure 4). These findings suggest that geographic variation in interaction strength among salt marsh plants is not always predictable based on latitude or aspects of the environment as anticipated by the SGH.

Variation among sites in interaction strengths, richness, and cover did not show latitudinal trends and could not be explained by measured environmental variables. Temperature, precipitation, soil salinity, and soil moisture explain geographic variation in species interactions in many salt marshes (Bertness & Ewanchuk, 2002; Keammerer & Hacker, 2013; Pennings et al., 2009; Whitcraft & Levin, 2007), yet were unrelated to differences among these sites in interaction strength or cover (Figure 4). Removal treatments had no significant effect on soil salinity or moisture, although these effects may have been more pronounced in the summer when we did not sample the experiment. More dramatic removals would likely have stronger effects on the environment as cover was reduced after removal treatments but remained high, ranging from 67% in a cool site to 133% in the northernmost site. Alternatively, the relationship between latitude and species interactions may only be apparent over even larger geographic scales (He et al., 2013). However, our sites varied considerably in temperature, precipitation, and salinity with maximum average site‐level salinities being 43% higher than the sites with the lowest salinities. This environmental gradient is comparable to the lower range of a 50%–75% increase in salinity that elicited changes in species interactions on the east coast (Bertness & Ewanchuk, 2002). Finally, other variables such as soil nutrients may explain the strength of competitive interactions (Bertness, Ewanchuk, & Silliman, 2002; Borer et al., 2014; Hautier, Niklaus, & Hector, 2009; Levine, Brewer, & Bertness, 1998); nutrient additions affect growth and species interactions of California salt marsh plants in species‐specific and context‐dependent ways (Morzaria‐Luna & Zedler, 2014; Ryan & Boyer, 2012). The factors responsible for the variable effects of species removal in our experiment remain unknown.

Community composition varied along the latitudinal gradient, unlike other community metrics. Community composition may be more sensitive to large‐scale patterns in temperature and precipitation. Despite latitudinal variation in composition, 84% of cover was made up of five species present across the whole range and the five southernmost sites had substantial overlap in composition (Figure 2; Table S1). Because nearby sites were more similar in composition, we might expect them to show similar interaction strengths. Alternatively, nearby sites may differ in interaction strengths because they have similar species composition and different environments, as was seen in New England salt marshes (Bertness & Ewanchuk, 2002), resulting in separate geographic trends in sites with distinct composition. Yet nearby sites and those that overlapped most in the species they contained were neither most similar nor most distinct in interaction strength (Figure 3; Table S1), suggesting that variation in interaction strength is not driven by changes in community composition.

Our results suggest that different environmental factors may shape interactions among salt marsh plants on the east and west coasts of North America. California marshes are more arid than east coast salt marshes which leads to higher soil salinities (Zedler, 1982). As a result, California salt marsh floras may be characterized by more salt‐tolerant species compared to those on the east coast, particularly in New England, explaining the lack of strong facilitation in this study (Pennings et al., 2003). Similarly, few cases of facilitation were seen in a study of interactions in Oregon marshes using several of the same species as in our study (Keammerer & Hacker, 2013). Thus, the species that occur in west coast salt marshes may tolerate greater salinity than those on the east coast, diminishing the importance of facilitation among species in the face of high salinity. Alternatively, the dominant plants may exert weaker effects on salinity on the west than the east coast.

We saw little evidence of facilitation by the dominant species, unlike in other systems (Figure 3; Bruno & Bertness, 2001; Stachowicz, 2001; Ellison et al., 2005). For example, in prairies, dominant species facilitate subordinate species in stressful conditions (Richardson et al., 2012). Similarly, in east coast salt marshes, the dominant *Spartina patens* facilitates other species by reducing salinity stress (Gedan & Bertness, 2010; Shumway & Bertness, 1992). We expected that *S. pacifica* might be able to facilitate subordinate species as it has been shown to reduce temperatures and porewater salinity in a southern California salt marsh (Whitcraft & Levin, 2007). *S. pacifica* might also be capable of facilitating other species low in the marsh as it is tolerant to flooding and low oxygen conditions (Mahall & Park, 1976; Pennings & Callaway, 1992). However, its presence did not facilitate other species except very weakly at Carpinteria. In fact, if some belowground competition with removed plants continued after *S. pacifica* removal, that may have even weakened our measurable competition effect, indicating that the dominant *S. pacifica* has net competitive interactions with subordinate species. This suggests that conditions other than the typical salinity and inundation may be more important to subordinate species fitness in these marshes.

Interestingly, the only facilitation we observed was of the dominant by the subordinate species. This is not the expected trend, yet there are other cases in which the subordinate species facilitate the dominant. The competitively dominant species in another salt marsh study was facilitated by subordinate species in a stressful environment (Bertness, 1991). The subordinate species were tolerant of high salinity conditions, allowing them to colonize stressful areas and make them more hospitable for the dominant species by modifying soil conditions. In our experiment, the common subordinate species such as *J. carnosa* and *D. spicata* were generally lower to the ground and grew more densely than the dominant *S. pacifica*; they may facilitate *S. pacifica* by shading the soil, modifying soils and microclimate. This may be why facilitation occurred only at the first sampling date when modification of the microclimate was likely most important as environmental conditions were most stressful due to the sudden loss of plant cover. A common garden experiment also found that *J. carnosa* facilitated growth of *S. pacifica* (Noto & Shurin, 2016). Thus, in addition to facilitation by dominant species, this study shows that subordinate species can also exert positive effects on performance of the dominant species.

It is worth noting that when using biomass to determine interaction strength, neither *S. pacifica* nor subordinate species had competitive or facilitative effects. This may be because too small a portion of the plot was destructively sampled or because by the end of the experiment, plants had grown back despite removals and dramatic effects were no longer apparent. This would be consistent with the strongest facilitation by subordinates being measured at the first time point (Fig. S2).

Geographic variation in interaction strength may be influenced by adaptive genetic differences among populations as well as environmental conditions. Populations may differ as a result of adaptation to local conditions which can affect the strength and direction of their interactions (Espeland & Rice, 2007). In a previous study, we found that source population affected interaction strength between *S. pacifica* and *J. carnosa* more than precipitation (Noto & Shurin, 2016). The present study included those two species and took place at the six sites from which plants were collected in the previous study, suggesting that variation among populations could also play a role in this experiment. Site‐based variation in the effect of *S. pacifica* on subordinate species observed in this experiment may be explained by genetic differences among populations, rather than variation in environmental context.

Our results support previous studies that have found idiosyncratic changes in interaction strength with latitude or environment. In arid grasslands and shrublands, changes in precipitation did not affect the strength of competition between the dominant and subordinate species (Peters & Yao, 2012). Theories that herbivory is stronger at low latitudes have also found mixed empirical support (Moles et al., 2011). For instance, in mangroves in the Western Hemisphere, herbivory was greatest at the most temperate location and least at an intermediate site (Feller et al., 2013). Species interactions may not show consistent geographic trends but instead be shaped by the interaction between climate, local conditions, and population variation in response to the environment.

Our study suggests that geographic variation in interaction strength may depend on conditions in local sites more than large‐scale gradients in temperature or precipitation. Variation in species interactions may be better explained by local factors such as soil fertility, consumer species, or genetic differences rather than large‐scale variation in climate (Borer et al., 2014; Hautier et al., 2009; He, Altieri, & Cui, 2015; Noto & Shurin, 2016). This suggests that the effects of climate on species interactions may be unpredictable due to interactions between climate and local‐scale environmental features. Theories about latitudinal variation in interaction strength may therefore be difficult to generalize to different regions because of local‐scale differences in the environment or species traits.

## CONFLICT OF INTEREST

None declared.

## AUTHOR CONTRIBUTIONS

AEN and JBS conceived and designed the experiments. AEN performed the experiments and data analysis. AEN wrote the manuscript and JBS provided editorial advice.
