# Supplementary material for: Interactions among salt marsh plants vary geographically but not latitudinally along the California coast
Source: Ecol Evol. 2017 Jul 14;7(16):6549–58. doi: 10.1002/ece3.3191 (PMC5574795; doi:10.1002/ece3.3191)
Supplement: Supplementary file 1 [file ECE3-7-6549-s001.docx]

**Supplementary Materials**

MS Title: **Interactions among salt marsh plants vary geographically but not latitudinally along the California coast**

Akana E. Noto^1^ and Jonathan B. Shurin^1^

^1^Section of Ecology, Behavior and Evolution, University of California, San Diego, La Jolla, California, USA.

Table A1: Species presence across sites over all sampling dates.

|  | TJ | KF | CAR | ELK | TOM | HUM |
| --- | --- | --- | --- | --- | --- | --- |
| *Salicornia pacifica* | X | X | X | X | X | X |
| *Distichlis spicata* |  | X | X | X |  | X |
| *Distichlis littoralis* |  |  | X |  |  |  |
| *Jaumea carnosa* | X | X | X | X | X | X |
| *Batis maritima* | X | X |  | X |  |  |
| *Frankenia salina* | X | X | X | X |  |  |
| *Limonium californicum* |  | X | X |  | X |  |
| *Triglochin concinna* |  | X | X |  | X |  |
| *Cuscuta salina* |  | X | X |  |  |  |
| *Salicornia bigelovii* |  | X |  |  |  |  |
| *Spartina foliosa* |  | X |  |  |  |  |
| *Suaeda esteroa* |  | X |  |  |  |  |
| *Spergularia* sp. |  |  | X |  | X |  |

Table A2: Percent cover and biomass (g) of *S. pacifica* and subordinate species in each removal treatment at each site (mean ± SE). Percent cover values are averaged across all sampling dates while biomass was only collected at the end of the experiment.

|  |  | Site | | | | | |
| --- | --- | --- | --- | --- | --- | --- | --- |
|  |  | TJ | KF | CAR | ELK | TOM | HUM |
| Control | *S. pacifica* cover | 48.6 ± 3.2 | 45.8 ± 2.9 | 34.9 ± 2.7 | 59.5 ± 3.8 | 72.5 ± 4.2 | 49.6 ± 2.7 |
|  | *S. pacifica* biomass | 21.8 ± 7.4 | 46.4 ± 14.7 | 23.6 ± 6.5 | 66.7 ± 22.8 | 52.7 ± 5.8 | 37.8 ± 7.9 |
|  | Sub cover | 71.3 ± 3.9 | 59.5 ± 4.5 | 95.2 ± 3.3 | 48.1 ± 5.1 | 34.5 ± 2.9 | 91.5 ± 2.9 |
|  | Sub biomass | 92.4 ± 17.6 | 50.9 ± 16.3 | 73.9 ± 4.7 | 18.6 ± 4.9 | 12.0 ± 3.3 | 78.7 ± 6.9 |
| *S. pacifica* removal | *S. pacifica* cover | 0.85 ± 0.2 | 2.6 ± 0.5 | 2.1 ± 0.5 | 4.6 ± 1.4 | 4.0 ± 0.5 | 3.3 ± 0.5 |
|  | *S. pacifica* biomass | 0.2 ±0.2 | 0.15 ± 0.07 | 1.2 ± 0.5 | 1.4 ± 1.2 | 0.2 ± 0.06 | 1.3 ± 0.2 |
|  | Sub cover | 88.0 ± 2.7 | 73.8 ± 3.7 | 93.4 ± 4.8 | 59.2 ± 6.1 | 55.4 ± 4.3 | 112.1 ± 2.9 |
|  | Sub biomass | 68.5 ± 12.4 | 52.5 ± 3.1 | 73.4 ± 11.9 | 32.1 ± 10.4 | 24.7 ± 7.3 | 81.2 ± 5.6 |
| Sub removal | *S. pacifica* cover | 48.3 ± 4.2 | 38.3 ± 2.0 | 35.3 ± 2.9 | 57.8 ± 4.0 | 63.5 ± 2.8 | 50.0 ± 1.8 |
|  | *S. pacifica* biomass | 31.2 ± 8.3 | 36.61 ± 6.5 | 21.7 ± 9.1 | 82.8 ± 22.8 | 51.2 ± 7.8 | 38.3 ± 6.6 |
|  | Sub cover | 50.0 ± 4.7 | 64.2 ± 3.7 | 76.3 ± 5.1 | 37.7 ± 5.2 | 36.3 ± 2.7 | 83.4 ± 3.3 |
|  | Sub biomass | 53.3 ±15.9 | 37.3 ± 4.9 | 62.3 ± 7.2 | 14.6 ± 7.6 | 8.6 ± 2.5 | 82.5 ± 10.2 |


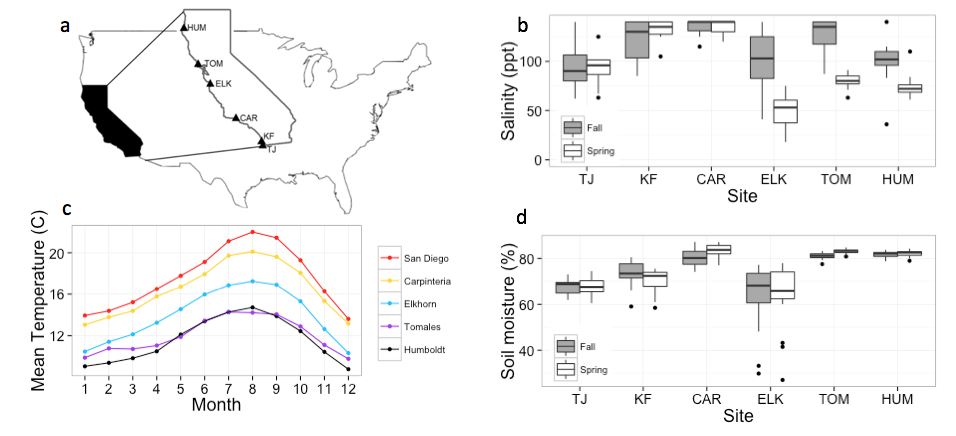


Fig. A1: a. Site locations, b. soil salinities, c. mean temperatures over the course of the year and d. soil moisture in each site. Salinity and soil moisture were measured in fall (grey) and spring (white). In boxplots, boxes indicate the first and third quartiles, whiskers extend to 1.5*(interquartile range), and data beyond that are depicted as points to indicate outliers.


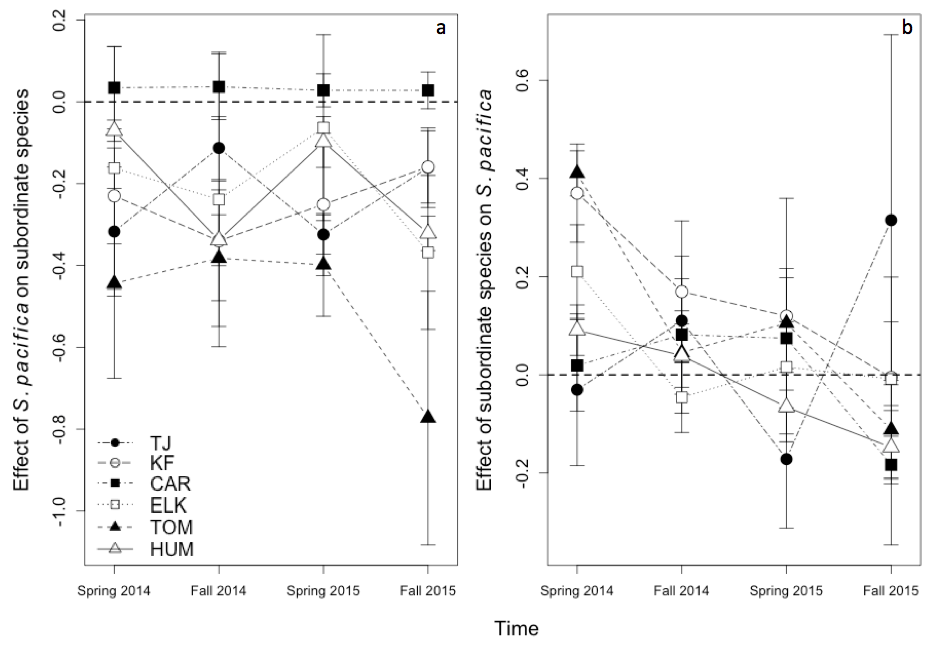


Fig. A2: a. Effect of *S. pacifica* on subordinate species and b. effect of subordinate species on *S. pacifica* over time by site. Positive values indicate facilitation and negative values indicate competition. Values are means ± SE.


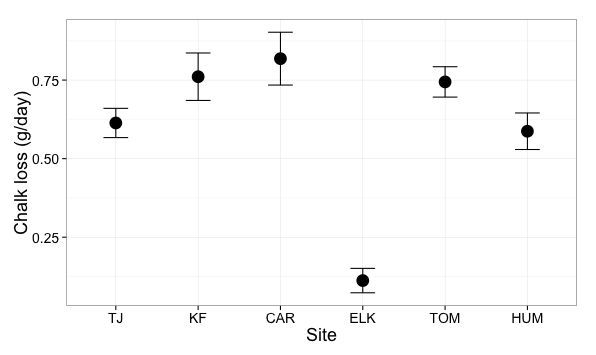


Fig. A3: Mass lost from chalk blocks (g/day) as a measure of erosion due to waves. Sites are listed from south (left) to north (right). Values are means ± SE.
